# Diffusion Capillary Phantom vs. Human Data: Outcomes for Reconstruction Methods Depend on Evaluation Medium

**DOI:** 10.3389/fnins.2016.00407

**Published:** 2016-09-07

**Authors:** Sarah D. Lichenstein, James H. Bishop, Timothy D. Verstynen, Fang-Cheng Yeh

**Affiliations:** ^1^Department of Psychology, Center for the Neural Basis of Cognition, University of PittsburghPittsburgh, PA, USA; ^2^Department of Neurological Sciences, University of VermontBurlington, VT, USA; ^3^Department of Psychology, Center for the Neural Basis of Cognition, Carnegie Mellon UniversityPittsburgh, PA, USA; ^4^Department of Neurological Surgery, University of PittsburghPittsburgh, PA, USA

**Keywords:** diffusion MRI, reconstruction, phantom, human brain, tractography

## Abstract

**Purpose:** Diffusion MRI provides a non-invasive way of estimating structural connectivity in the brain. Many studies have used diffusion phantoms as benchmarks to assess the performance of different tractography reconstruction algorithms and assumed that the results can be applied to *in vivo* studies. Here we examined whether quality metrics derived from a common, publically available, diffusion phantom can reliably predict tractography performance in human white matter tissue.

**Materials and Methods:** We compared estimates of fiber length and fiber crossing among a simple tensor model (diffusion tensor imaging), a more complicated model (ball-and-sticks) and model-free (diffusion spectrum imaging, generalized q-sampling imaging) reconstruction methods using a capillary phantom and *in vivo* human data (*N* = 14).

**Results:** Our analysis showed that evaluation outcomes differ depending on whether they were obtained from phantom or human data. Specifically, the diffusion phantom favored a more complicated model over a simple tensor model or model-free methods for resolving crossing fibers. On the other hand, the human studies showed the opposite pattern of results, with the model-free methods being more advantageous than model-based methods or simple tensor models. This performance difference was consistent across several metrics, including estimating fiber length and resolving fiber crossings in established white matter pathways.

**Conclusions:** These findings indicate that the construction of current capillary diffusion phantoms tends to favor complicated reconstruction models over a simple tensor model or model-free methods, whereas the *in vivo* data tends to produce opposite results. This brings into question the previous phantom-based evaluation approaches and suggests that a more realistic phantom or simulation is necessary to accurately predict the relative performance of different tractography reconstruction methods.

## Introduction

Diffusion MRI is an increasingly popular imaging approach for visualizing *in vivo* white matter architecture. It detects the movement of water, which is less constrained along the axons in the brain tissue than in the perpendicular direction. The diffusion signals can reveal such an anisotropy that can be used as a proxy for the general orientation of the fiber, making it possible to draw inferences about the microstructural properties of white matter pathways (Hagmann et al., 2006; Jones et al., 2013). This information has been applied to study normal white matter connectivity as well as to elucidate the neural basis of various forms of clinical pathology (Seizeur et al., 2012; Jones et al., 2013), including multiple sclerosis (Filippi and Rocca, 2011), stroke (Sotak, 2002), Alzheimer's disease (Amlien and Fjell, 2014), and a variety of neuropsychiatric conditions such as schizophrenia, mood, and anxiety disorders (White et al., 2008).

While diffusion MRI holds great promise for characterizing structural connectivity of the brain, there is substantial methodological heterogeneity that can pose challenges for interpreting findings across studies. In particular, different reconstruction methods vary in the degree to which they can capture complex patterns of water diffusion within voxels. Therefore, it is important to delineate the relative strengths and weaknesses of different diffusion imaging analysis techniques in order to help researchers select the most appropriate methods when designing studies of white matter architecture.

Model-based reconstruction approaches parameterize the diffusion signal using either a tensor model, i.e., diffusion tensor imaging (DTI) (Basser et al., 1994), ball-and-sticks model (BSM; Behrens et al., 2003), kurtosis model (Jensen et al., 2005), or a response function (Tournier et al., 2008) to calculate the fiber directions and assume that specific diffusion geometries arise from axonal structures within a voxel. On the other hand, model-free reconstruction methods, such as diffusion spectrum imaging (DSI) (Wedeen et al., 2005), q-ball imaging (Tuch, 2004), and generalized q-sampling imaging (GQI) (Yeh et al., 2010), use nonparametric approaches to estimate the orientation distribution function (ODF) of diffusion, also known as the diffusion ODF (dODF). These methods do not assume a specific model, and the local maxima in the dODFs are used to guide fiber tracking (Wedeen et al., 2012; Yeh et al., 2013).

To evaluate the performance of different methods, diffusion algorithm development and imaging parameter optimization is an active field of study with several seminal studies exhaustively comparing diffusion reconstructions in phantom data sets. Fillard and colleagues, the creators of the Fibercup phantom utilized in this investigation, developed the phantom for a tractography competition comparing the results of 10 novel tractography algorithms (Fillard et al., 2011). In another report, Daducci and colleagues investigated 20 reconstruction approaches across several classes of diffusion algorithms in two distinct phantom data sets, including many classically implemented options available in freely available software (Daducci et al., 2014). Additionally, Côté et al. recently developed an online evaluation method and compared 57, 096 different diffusion imaging analysis pipelines using the Fibercup dataset (Côté et al., 2013). These studies have highlighted how model-based reconstruction approaches, particularly more complicated models such as constrained spherical deconvolution (Tournier et al., 2008) and the ball-and-sticks model (BSM; Behrens et al., 2007), have higher angular resolution in the reconstructed fiber orientations than model-free methods (Tournier et al., 2008; Fillard et al., 2011; Daducci et al., 2014) and emphasized that methods with higher angular resolution should lead to more accurate mapping of brain connections *in vivo*. However, a recent tractography competition revealed a dramatically different result (http://www.tractometer.org/ismrm_2015_challenge/), showing that a simple tensor model and model-free approaches, such as GQI, provided more accurate mapping, whereas methods with a much higher angular resolution did not necessarily yield more valid connections. This leads to a critical question on whether current diffusion phantoms built by capillary tubes are representative of diffusion characteristics observed in *in vivo* white matter fascicles.

Here we use publicly available phantom data and *in vivo* human data to evaluate the relative performance of a simple tensor model, a more complicated ball-and-sticks model, and model-free reconstruction methods. In particular, we hypothesize that the capillary diffusion phantom can discriminate the performance between reconstruction approaches in the same way as is observed in established human pathways. To consider the influence of the diffusion sampling schemes, we selected methods that can be equally applied to both single *b*-value and multiple *b*-value acquisitions. This includes model-based methods such as DTI and ball-and-sticks as well as model-free methods such as GQI, and DSI. We focused on phantom pathways that are representative of the complex fiber geometry of the human centrum semiovale in order to provide a comparable benchmark test between phantom and human data. Additional consideration for sampling scheme selection was made based on implementation in commonly available freeware image processing packages.

## Materials and methods

### Phantom study

#### Phantom design

The Fibercup diffusion MR phantom (Poupon et al., 2008; Fillard et al., 2011) is the only currently publicly available phantom data with complex fiber geometry. It consists of seven distinct bundles of hydrophobic acrylic fibers contained within a MRI-compatible cylindrical container. The phantom was designed to simulate a coronal section of the human brain and includes a number of different anatomically relevant crossing and kissing fiber configurations with different curvatures. In particular, the upper portion of the Fibercup phantom contains several crossing pathways that are intended to approximate the complex fiber-crossing geometry of the human centrum semiovale. Additionally, the straight arm of the phantom with minimal fiber crossings is anatomically indicative of the human corticospinal pathway. Together, these geometries provide an ideal analog to compare the performance of different diffusion analysis methods for resolving crossing fibers between the Fibercup phantom and *in vivo* human data. Phantom fibers are 20 μm in diameter, distributed evenly, and compressed, resulting in approximately 1900 fibers/mm^2^ throughout the device, including points of intersection.

#### Phantom image acquisition and reconstruction

Diffusion-weighted images were acquired on a Siemens 3T Tim Trio MRI scanner at the NeuroSpin center using a 12-channel head coil, and a single-shot diffusion-weighted twice refocused spin echo planar pulse sequence. Acquisition parameters included spatial resolution = 3 mm isotropic, FOV = 19.2 cm, matrix 64 × 64, slice thickness TH = 3 mm, read bandwidth = 1775 Hz/pixel, partial Fourier factor 6/8, parallel reduction factor GRAPPA = 2, repetition time TR = 5 s, 2 repetitions. A total of 64 diffusion directions were obtained with multiple *b*-values = 650/1500/2000 s/mm^2^, corresponding to the echo times TE = 77/94/102 ms respectively. Three slices were acquired. The image data are publicly available at https://www.nitrc.org/frs/?group_id=627.

Diffusion tensors were calculated using DSI Studio (http://dsi-studio.labsolver.org), and fractional anisotropy (FA) was used to guide the deterministic fiber tracking (Yeh et al., 2013). In addition to diffusion tensor, the ball-and-sticks model was calculated using *bedpost* in the FSL Diffusion Toolkit (Behrens et al., 2007) (http://fsl.fmrib.ox.ac.uk/fsl/). GQI (Yeh et al., 2010) was calculated in DSI Studio (http://dsi-studio.labsolver.org) with a diffusion sampling length ratio of 1.5 and a maximum of 3 fibers resolved. Additionally, a second iteration of the GQI reconstruction was performed that applied a deconvolution algorithm to sharpen the ODFs, which has been shown to improve the angular resolution of non-tensor based reconstruction techniques (Yeh et al., 2011). In the current analyses, a regularization parameter of 7 was applied as suggested by the original study.

Deterministic fiber tracking was performed with DSI Studio (http://dsi-studio.labsolver.org). A generalized deterministic tracking algorithm (Yeh et al., 2013) was used and two million seeds were randomly generated at subvoxel positions and random orientations. Tracking progression continued with a step size of 0.5 mm, and each step was weighted by 40% of the previous direction to smooth the tracks. Otsu's method (Otsu, 1979) was used to set the anisotropy thresholds for the DTI (FA) and GQI (QA) reconstructions. In the ball-and-sticks model, the fiber ratio was used as the stopping criteria. Because the ball-and-sticks analysis has been traditionally applied with probabilistic tractography (Behrens et al., 2007), there is no established method for setting a fiber ratio threshold for deterministic tractography with this approach. Therefore, in order to avoid biasing our evaluation of the ball-and-sticks reconstruction by selecting a single fiber ratio threshold, we selected two different thresholds based on a comparison of phantom fiber tracking results with a range of different thresholds from 0.01 to 0.05. The 0.01 threshold yielded the greatest number of streamlines, whereas thresholds above 0.03 failed to produce any streamlines. Therefore, the 0.01 and 0.03 fiber ratio thresholds were selected for the ball-and-sticks analysis. Tracking was terminated if the anisotropy/fiber ratio of the next step fell below the assigned threshold, or exceeded an angular threshold of 65°. Streamline lengths were constrained to 1–150 mm. Ten randomized iterations of the fiber tracking were performed in order to evaluate the variability of streamlines in addition to comparing their accuracy to absolute fiber measurements. Mean values were calculated across iterations of each reconstruction method for across method comparisons.

#### Phantom fiber length analysis

**Figure 1:**
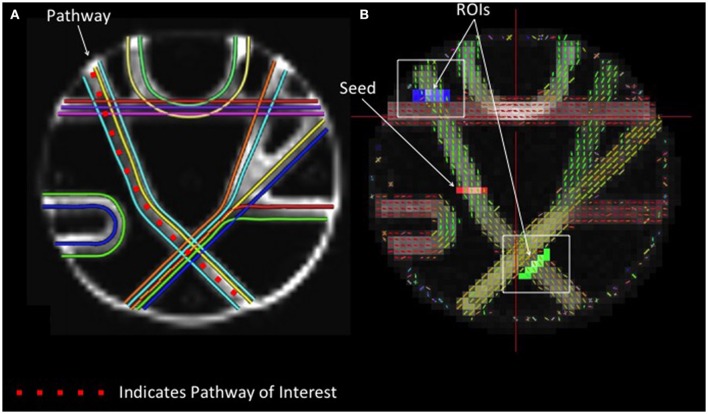
**Phantom Fiber Length Analysis. (A)** (Left) Illustrates ground truth fiber orientations within each segment of the Fibercup phantom. Each color represents a distinct fiber pathway. **(B)** To test the accuracy of each reconstruction scheme for estimating fiber length, we placed a seed in one pathway and defined regions of interest immediately following the fiber crossings (blue and green) to isolate only those streamlines that traverse the crossings. For resulting streamline length estimates refer to **Figure 4**. ROIs: regions of interest.

The arm of the Fibercup phantom with the least fiber crossings was selected to examine the accuracy of streamline length estimates obtained with different reconstruction methods. A seed region was placed at the center of the pathway, and fiber tracking was initiated from this point. In order to attenuate the confounding effect of crossing fibers on our measure of streamline length, ROIs were drawn beyond each intersecting fiber to isolate those streamlines that successfully navigated these crossings (Figure 1). The distributions of streamline length estimates derived from different reconstruction methods were directly compared. Each streamline length was multiplied by its probability to approximate the area under the curve to quantify the modal streamline length estimate for each reconstruction approach. Univariate analysis of variance (ANOVA) was used to compare streamline length measurements across reconstruction methods, and Bonferroni post-hoc tests were utilized to determine which pairwise differences were significant.

#### Phantom fiber crossing analysis

**Figure 2:**
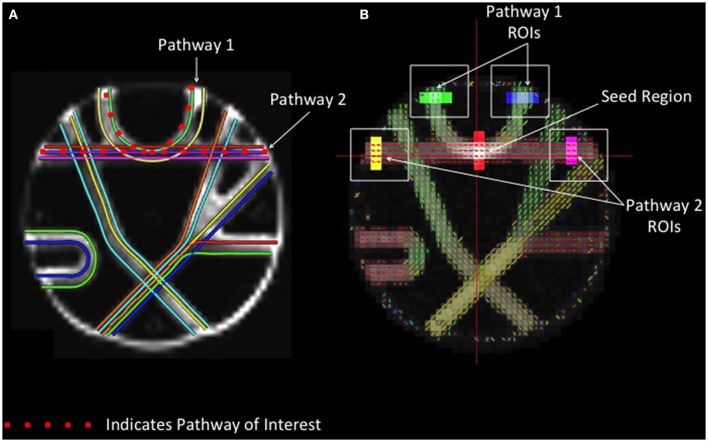
**Phantom Fiber Crossing Analysis. (A)** The dotted red lines define two pathways of interest in the Fibercup phantom, representative of the corpus callosum architecture. The first pathway (Pathway 1) is indicative of cortical “U” fibers and the second pathway (Pathway 2) represents traversing callosal fibers. **(B)** Regions of interest were defined at the end of each pathway (shown in yellow, pink, blue, and green) and single seed region (red) was placed at the center of pathways 1 and 2. The accuracy of each reconstruction method was assessed based on the number of streamlines passing through the seed region and both endpoints of the respective pathways (refer to **Figure 5**). ROIs: regions of interest.

In order to compare the performance of different reconstruction methods for resolving crossing fibers, a seed region was defined at the intersection between two crossing tracts in the upper portion of the phantom with geometry similar to the human centrum semiovale (Figure 2). Fiber tracking in these two known pathways was conducted, and regions of interest were drawn manually at each end of both pathways. Accuracy was assessed by quantifying the number of streamlines that passed through the seed region and both endpoints of the respective pathways.

### Human study

#### Human participants

Fourteen healthy adults (5 men, 9 women, mean age = 27.2 ± 3.8, age range 21–33) participated in the study. Subjects were screened for any contraindications to MRI scanning, and provided informed consent prior to participation. All procedures were approved by the Institutional Review Board at Carnegie Mellon University.

#### Human imaging acquisition and reconstruction

Diffusion spectrum imaging data were acquired on a Siemens 3T Verio scanner, in the Scientific Imaging for Brain Research Center on the Carnegie Mellon University campus, using a 32-channel coil and a twice-refocused spin-echo echo planar imaging (EPI) diffusion sequence. Acquisition parameters included spatial resolution = 2.4 mm isotropic, TR = 9916 ms, TE = 157 ms, in-plane resolution = 2.4 mm. A total of 512 diffusion directions were obtained using a half-sphere scheme with a maximum *b*-value of 5000 s/mm^2^.

Diffusion tensor, ball-and-sticks model, and GQI were conducted using the same parameters as the phantom study. DSI reconstruction was also performed with ODFs with 642 sampling directions, a diffusion sampling length ratio of 1.5, a hanning filter of 16, and 3 fibers resolved. A second iteration of the DSI reconstruction was performed using a deconvolution algorithm with a regularization parameter of 7.

#### Human fiber length analysis

The same streamline (Euler) deterministic tracking algorithm was used as in the phantom study and 100,000 tracts were generated using random whole brain seeding at subvoxel positions and in random orientations. Tracking progression continued with a step size of 0.5 mm, and each step was weighted by 40% of the previous direction to smooth the tracks. Otsu's method was used to set the anisotropy thresholds for the DTI (FA), DSI (QA), and GQI (QA) reconstructions, and two different fiber ratio thresholds were selected for the ball-and-sticks analysis, 0.01 and 0.03, as described above. Tracking was terminated if the anisotropy of the next step fell below the assigned threshold, or exceeded an angular threshold of 65°. Streamline lengths were constrained to 1–160 mm. In the ball-and-sticks model, the fiber ratio was used instead of anisotropy as the stopping criteria.

For each reconstruction method, the distribution of streamline length estimates for each subject was generated and means and standard deviations were calculated. Modal streamline length measurements were estimated by multiplying each streamline length by its probability. ANOVA analyses were used to compare modal streamline length estimates derived with different reconstruction methods and Bonferroni *post-hoc* tests were conducted to determine which pairwise comparisons were significant.

#### Human fiber crossing analysis

In order to examine the accuracy of different methods for navigating complex fiber crossings, we compared the performance of each reconstruction method for recovering streamlines passing through the centrum semiovale, a region where several prominent white matter tracts intersect (Tuch et al., 2005; Schmahmann and Pandya, 2006; Jones, 2008; Sotiropoulos et al., 2013). Previous research has demonstrated that poor reconstructions of fiber crossings results in biases that tend to resolve only a single pathway or section of a pathway (Farquharson et al., 2013). Although corpus callosum fibers are known to terminate throughout the frontal cortex, poor resolution of crossing fibers in the centrum semiovale creates a bias toward the medial callosal U-shaped fibers due to an inability to capture more lateral streamlines that traverse the centrum semiovale crossing. Here, we evaluated whether different reconstruction methods differ in their ability to capture the lateral spread of corpus callosum pathways by comparing the relative number of streamlines that terminate in different sections of the frontal cortex.

Tractography for the fiber crossing analysis was conducted with a streamline (Euler) deterministic tracking algorithm. One million seeds were placed at random subvoxel positions throughout the brain. Tracking was initiated in random orientations, and was propagated with a step size of 1.2 mm and smoothing of 0.4. Anisotropy thresholds were determined using Otsu's method for reconstructions other than the ball-and-sticks, for which two thresholds were set at 0.01 and 0.03. Streamlines were terminated when the anisotropy at the subsequent step fell below the assigned threshold or the turning angle exceeded 60°.

**Figure 3:**
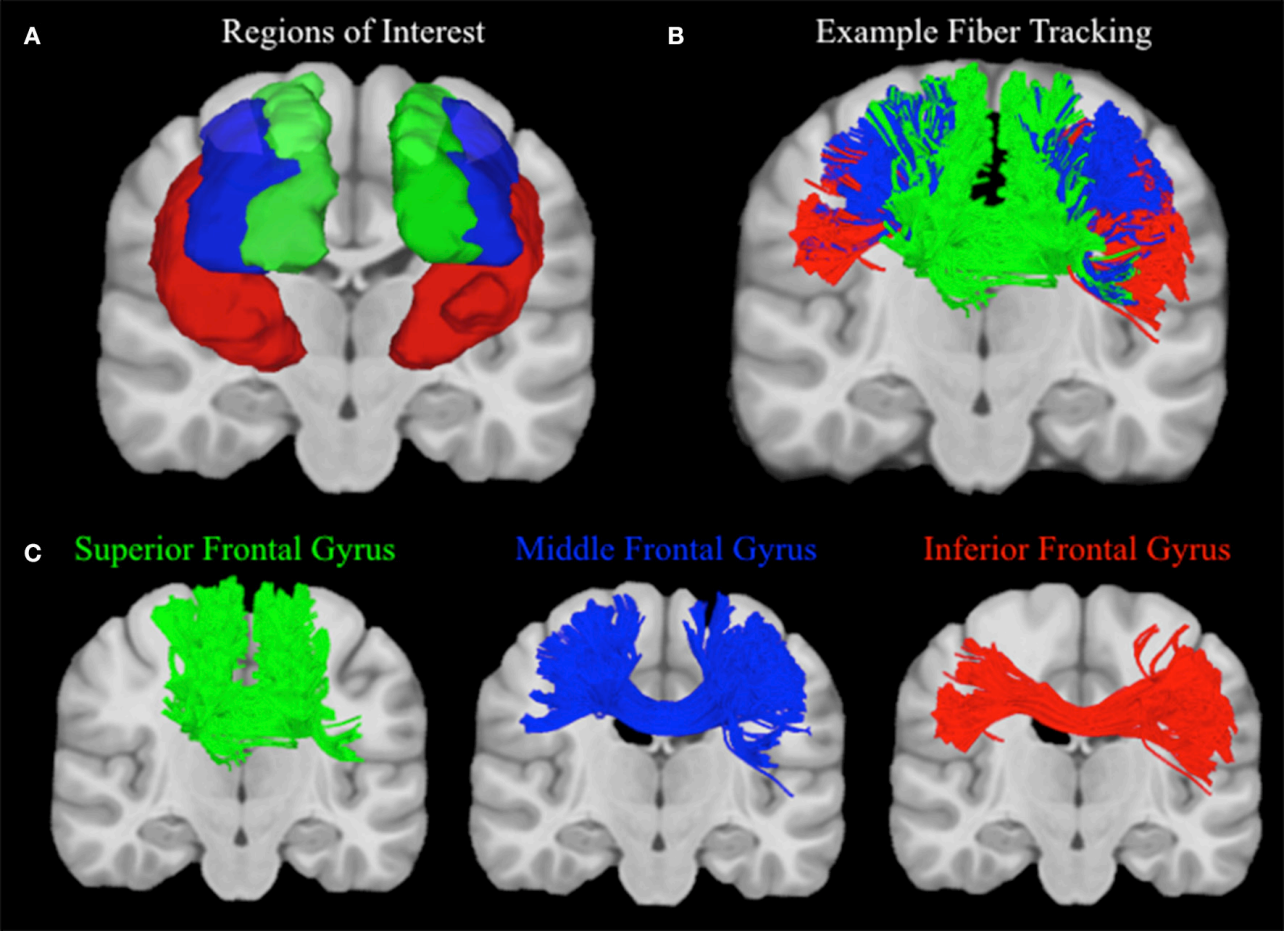
**Human Fiber Crossing Analysis. (A)** Depicted in a coronal plane, the automated anatomic labeling AAL atlas (Tzourio-Mazoyer et al., 2002) was used to define three topographical regions: the left and right superior frontal gyrus (Green), middle frontal gyrus (Blue), and inferior frontal gyrus (IFG) operculum, IFG triangularis, and the IFG orbitalis, which were merged to create one ROI for the inferior frontal gyrus (red). **(B)** A single subject was used to illustrate the topography of corpus callosum fibers connecting the left and right superior, medial, and inferior frontal gyri, respectively. Single-subject data were reconstructed using diffusion spectrum imaging and deterministic (Euler) fiber tracking was conducted in native space. One lakhs tracts were generated connecting the right and left hemispheres of the superior, medial, and inferior frontal gyri, respectively, using random whole brain seeding at subvoxel positions and in random orientations. Tracking progression continued with a step size of 0.5 mm, and each step was weighted by 40% of the previous direction to smooth the tracks. Otsu's method was used to set the anisotropy (QA) threshold, and tracking was terminated if the anisotropy of the next step fell below the assigned threshold, or exceeded an angular threshold of 65°. Streamline lengths were constrained to 1–160 mm. **(C)** In order to clearly illustrate the topography of each group of corpus callosum fibers, fibers connecting left and right superior, medial, and inferior frontal gyri are visualized separately.

Connectivity matrices for each participant were constructed using frontal cortex ROIs from the automated anatomic labeling (AAL) atlas (Tzourio-Mazoyer et al., 2002), including the left and right superior frontal gyrus (SFG), middle frontal gyrus (MFG), inferior frontal gyrus (IFG) operculum, IFG triangularis, and the IFG orbitalis. The number of streamlines that connect each hemisphere of the superior, middle, and inferior frontal gyrus ROIs were extracted from each connectivity matrix, and values from the three regions of the inferior frontal gyrus were summed (see Figure 3). Ratios of the number of SFG, MFG, and IFG streamlines relative to the total number of streamlines were calculated so although an exact streamline count is unknown, higher ratios of MFG and IFG streamlines would be indicative of better lateral coverage. Therefore, more accurate reconstruction methods should estimate greater proportions of streamlines in the medial and inferior regions of the frontal cortex, whereas less successful reconstructions will show a greater bias toward the superior, u-shaped fibers.

In order to quantitatively compare between reconstruction methods, a 7 × 3 ANOVA was conducted to assess the effects of reconstruction method and ROI on the ratio of streamlines resolved. In order to evaluate whether the application of a deconvolution algorithm improves lateral coverage, an additional analysis was conducted within the DSI and GQI data using a 2 × 2 ANOVA to test the effect of reconstruction method and deconvolution algorithm on the ratio of streamlines recovered for each ROI individually. Where significant group differences were found, follow-up analyses were conducted to determine which pairwise comparisons were significant.

## Results

### Phantom study results

#### Phantom fiber length results

Fiber tracking with the DTI reconstruction was unable to detect any streamlines passing through the long leg of the phantom that navigated both crossings. Means and standard deviations for number of streamlines detected with each of the other reconstruction methods are: ball-and-sticks (0.01 fiber ratio threshold) 43,320.2 ± 224.96; ball-and-sticks (0.03 fiber ratio threshold) 10,988.5 ± 141.84; GQI 213 ± 15.76; GQI with deconvolution 22,942.8 ± 166.33. Analysis of variance for the four reconstruction methods that were able to detect streamlines within this arm of the phantom demonstrated that there is a significant group difference in the number of streamlines resolved with different reconstruction methods [*F*_(3, 36)_ = 138362.57, *p* < 0.001]. *Post-hoc* pairwise comparisons show that each reconstruction method yields a significantly different number of tracts relative to each of the other reconstruction methods (all *p* < 0.001).

**Figure 4:**
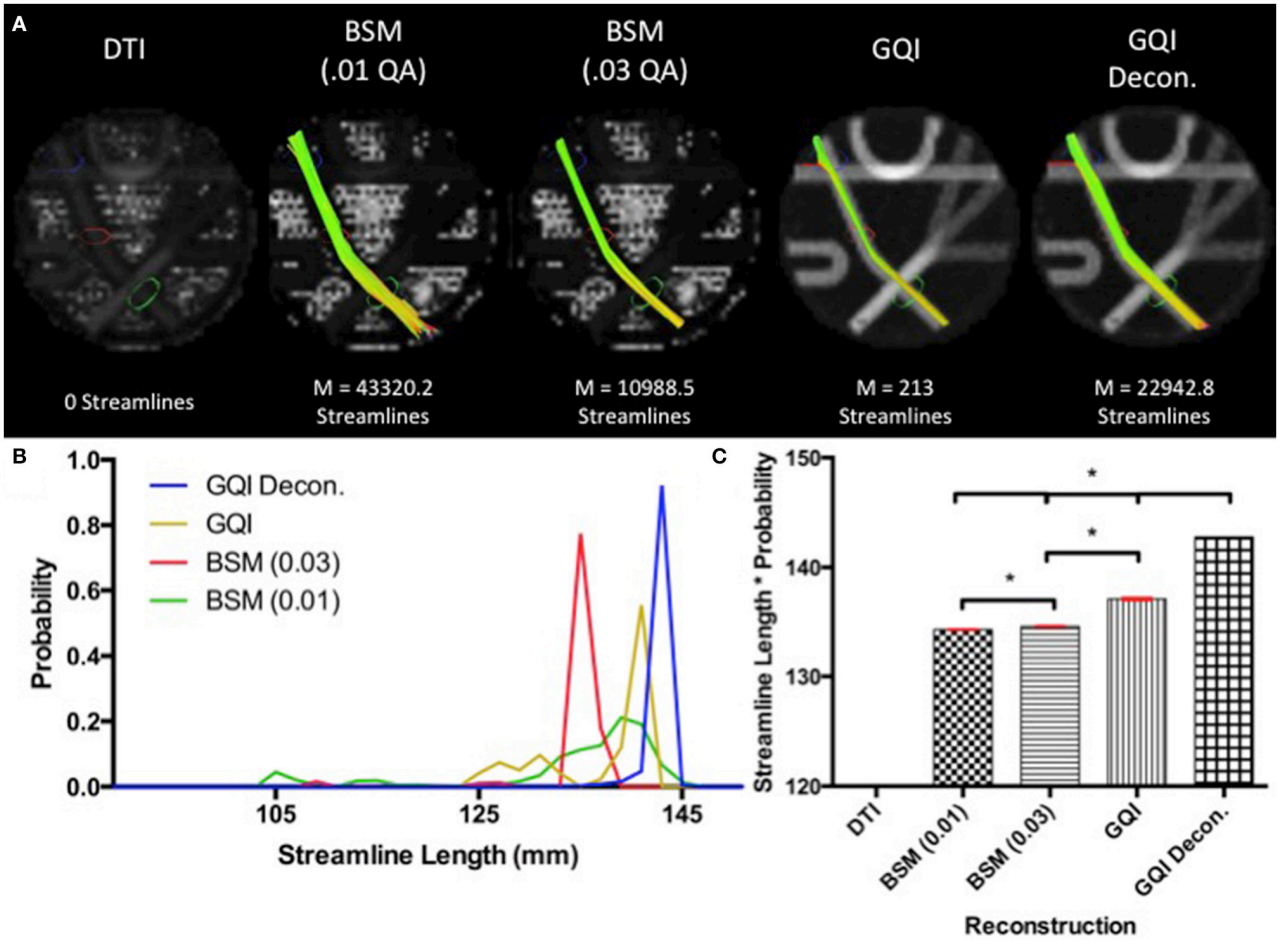
**Phantom Fiber Length Results**. **(A)** Example fiber tracking and the mean number of streamlines are represented for each reconstruction method in the Fibercup phantom. Fiber tracking with the DTI reconstruction was unable to detect any streamlines that navigated both crossings. **(B)** Distribution of streamline length estimates for each reconstruction method. GQI reconstructions produced longer and less variable streamline estimates compared to the ball-and-sticks model (BSM 0.01, BSM 0.03). **(C)** To quantitatively evaluate length estimates, the streamline lengths were multiplied by the probability. Each reconstruction method yields significantly different modal streamline length estimates relative to each other reconstruction method. DTI, diffusion tensor imaging; GQI, generalized q-sampling imaging; decon, deconvolution; M, mean; BSM, ball-and-sticks model. ^*^Significance of at least *p* < 0.05.

The distribution of streamline length estimates demonstrates that the ball-and-sticks model (0.01 fiber ratio threshold) yields the greatest number of streamlines within this pathway, but the length measurements were less accurate and more variable relative to the estimates generated with the GQI approaches (Figure 4). The GQI and GQI with deconvolution reconstructions produce fewer streamlines, but their length measurements are longer and had less variability. Analysis of variance demonstrates a significant group difference in modal streamline length estimates generated using the different reconstruction methods [*F*_(3, 36)_ = 7568.65, *p* < 0.001]. *Post-hoc* pairwise comparisons show that each reconstruction method yields significantly different length estimates relative to each of the other reconstruction methods (all *p* < 0.01).

#### Phantom fiber crossing results

**Figure 5:**
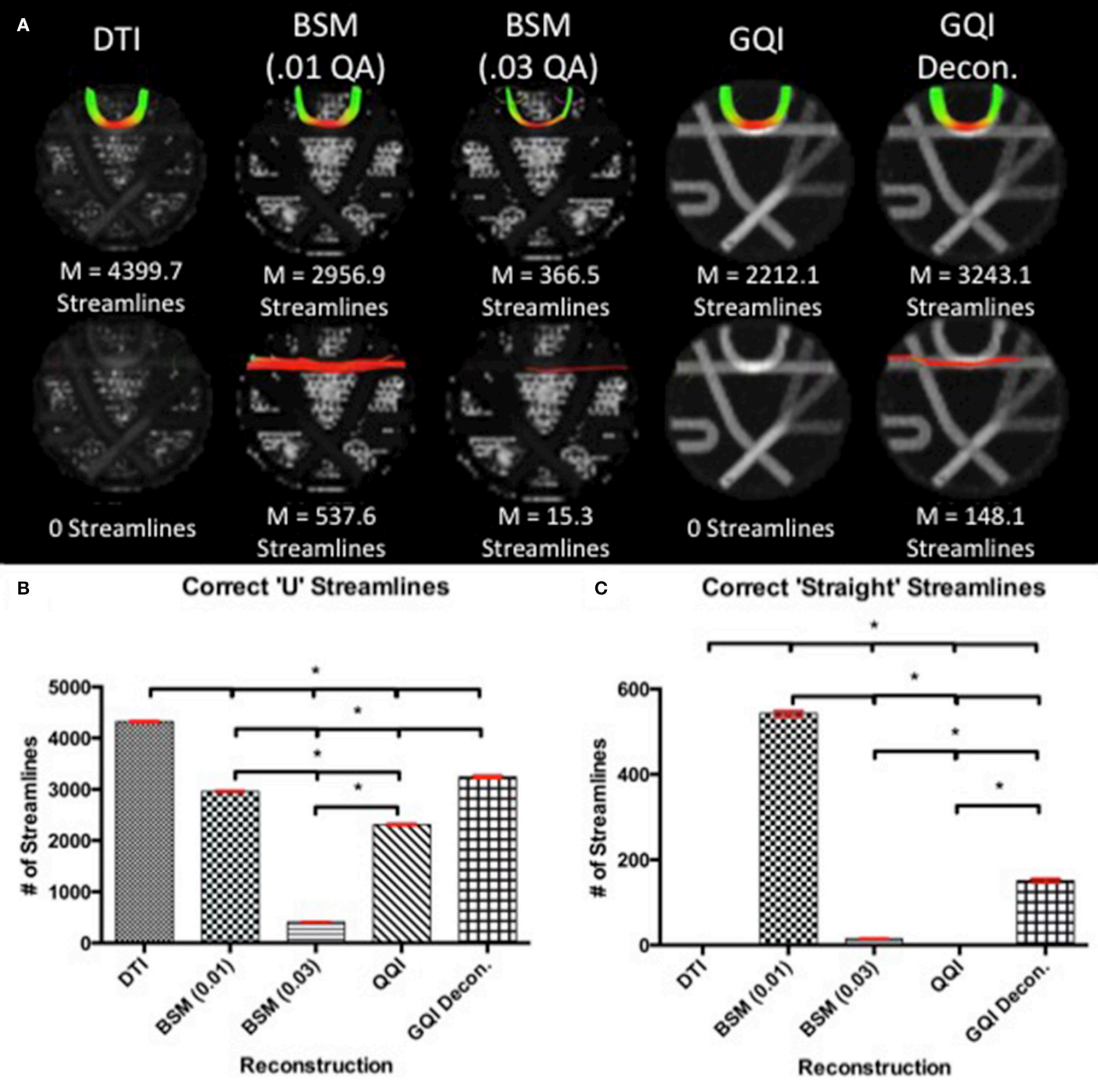
**Phantom Fiber Crossing Results. (A)** The top row illustrates the reconstruction tractography results for pathway 1, characteristic of cortical “U” fibers. The second row shows the tractography results across reconstruction methods in pathway 2 demonstrating successful traversal of the “U” fiber geometry. **(B)** Quantitative analysis based on the number of correct “U” streamlines demonstrated significant differences between all reconstruction methods with DTI providing the greatest number of correct streamlines. **(C)** Quantitative analysis based on the number of correct straight streamlines demonstrated that the ball-and-sticks reconstruction (0.01) generated the greatest number of correct streamlines while DTI and GQI were unable to produce any streamlines. DTI, diffusion tensor imaging; GQI, generalized q-sampling imaging; decon, deconvolution; M, mean; BSM, ball-and-sticks model. ^*^Significance of at least *p* < 0.05.

For pathway 1, the DTI reconstruction produces the greatest number of streamlines, detecting a mean of 4399.7 ± 46.76 streamlines, relative to the 2956.9 ± 42.53, 366.5 ± 14.80, 2212.1 ± 45.26, and 3243.1 ± 76.64 streamlines for ball-and-sticks (0.01 fiber ratio threshold), ball-and-sticks (0.03 fiber ratio threshold), GQI, and GQI with deconvolution, respectively (Figure 5). Analysis of variance indicates that there is a significant group difference in the number of tracts detected within pathway 1 [*F*_(4, 45)_ = 9179.71, *p* < 0.001]. *Post-hoc* pairwise comparisons show that each method yields a significantly different number of tracts relative to each other reconstruction (all *p* < 0.001).

In pathway 2, no streamlines were detected with the DTI or GQI reconstructions, whereas 537.6 ± 18.18 streamlines were identified with the ball-and-sticks model at a fiber ratio threshold of 0.01, 15.3 ± 3.09 streamlines were detected with the ball-and-sticks model at a 0.03 fiber ratio threshold, and 148.1 ± 15.70 were found using GQI with deconvolution. ANOVA results show a significant group difference in the number of tracts detected within pathway 2 [*F*_(4, 45)_ = 4538.48, *p* < 0.001]. *Post-hoc* pairwise comparisons show that the two ball-and-sticks reconstructions and GQI with deconvolution yield significantly different streamline measurements (all *p* < 0.05). DTI and GQI do not differ significantly because both methods failed to identify any tracts within this pathway.

### Human study results

#### Human fiber length results

**Figure 6:**
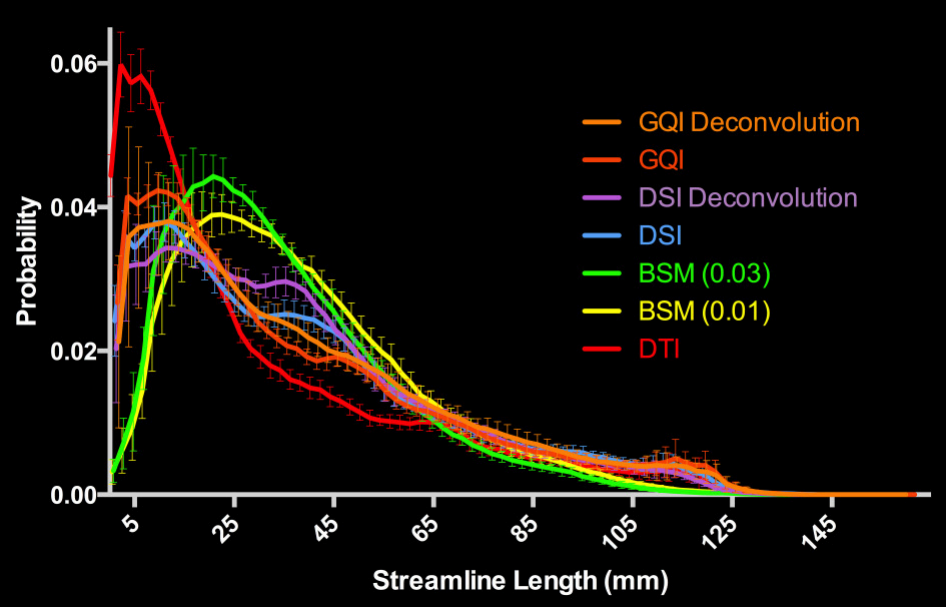
**Distribution of Human Streamline Length Estimates**. The DTI reconstruction produced the greatest number of streamlines <10 mm that is indicative of noise. The model-free (GQI, GQI Deconvolution, DSI, and DSI Deconvolution) and ball-and-sticks (BSM 0.01, BSM 0.03) reconstructions yielded a greater number of streamlines with increased length (>10 mm) suggestive of physiologically relevant dimensions. GQI, generalized q-sampling imaging; DSI, diffusion spectrum imaging; BSM, ball-and-sticks model; DTI, diffusion tensor imaging.

**Figure 7:**
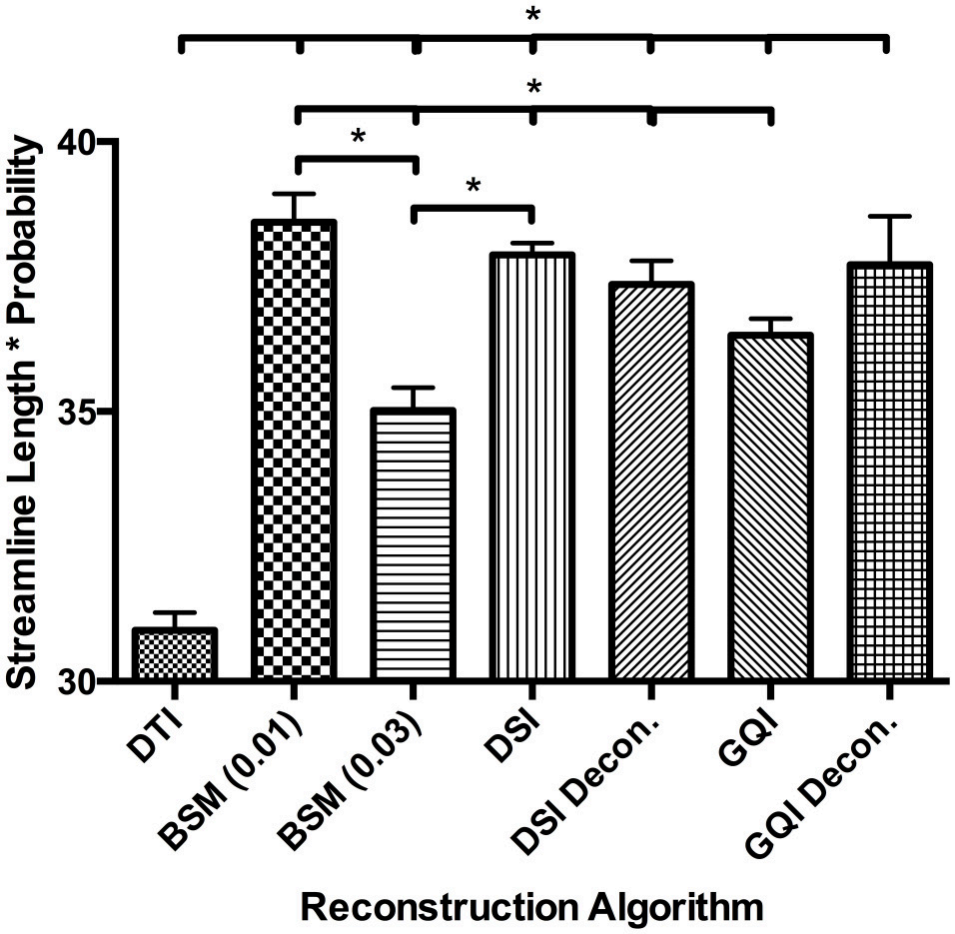
**Human Fiber Length Results**. To quantitatively evaluate length estimates the streamline lengths were multiplied by the probability. Results shown indicate significant differences between model-free (GQI, GQI Decon., DSI, and DSI Decon.) and ball-and-sticks (BSM 0.01, BSM 0.03) reconstructions. Additionally, single tensor (DTI) reconstruction showed a significantly lower modal streamline length compared to all other reconstruction methods tested. DTI, diffusion tensor imaging; BSM, ball-and-sticks model; DSI, diffusion spectrum imaging; GQI, generalized q-sampling imaging. ^*^Significance of at least *p* < 0.05.

For the human data, the distribution of streamline length estimates for each reconstruction method shows that the DTI approach produces the greatest quantity of small, noisy streamlines, whereas the ball-and-sticks model yields the fewest number of short streamlines, i.e., those shorter than 10 mm (Figure 6). Means and standard deviations for modal streamline length estimates acquired with each reconstruction method were: DTI 30.94 ± 1.23; ball-and-sticks (0.01 fiber ratio threshold) 38.51 ± 1.96; ball-and-sticks (0.03 fiber ratio threshold) 35.02 ± 1.58; DSI 37.9 ± 0.82; DSI with deconvolution 37.35 ± 1.63; GQI 36.41 ± 1.14; GQI with deconvolution 37.72 ± 3.36. Analysis of variance reveals a significant group difference in modal streamline length estimates generated using the different reconstruction methods [*F*_(6, 91)_ = 28.08, *p* < 0.001; Figure 7]. *Post-hoc* pairwise comparisons show that the DTI method yields significantly shorter length estimates relative to each other reconstruction (all *p* < 0.01). Additionally, there is a significant difference between the ball-and-sticks model at a 0.01 fiber ratio threshold and the ball-and-sticks model using a 0.03 fiber ratio threshold (*p* < 0.001). The ball-and-sticks reconstruction (0.03 QA) also differs significantly from the DSI, DSI with deconvolution and GQI with deconvolution methods (all *p* < 0.05). Streamline length estimates derived with the ball-and-sticks model at a 0.01 fiber ratio threshold, DSI or GQI reconstruction methods do not significantly differ from one another, with or without the application of the deconvolution algorithm.

#### Human fiber crossing results

Means and standard deviations for the total number of streamlines connecting the right and left superior, medial, and inferior frontal gyrus ROIs are presented in Table 1. The total number of streamlines differs significantly by reconstruction method [*F*_(6, 91)_ = 18.387, *p* < 0.001]. *Post-hoc* analyses revealed that this group difference is driven primarily by a greater number of streamlines detected with the DTI reconstruction and relatively fewer streamlines detected using the ball-and-sticks (0.01) approach; whereas the number of total streamlines does not differ between the ball-and-sticks reconstruction at the 0.03 fiber ratio threshold, DSI, or GQI, regardless of deconvolution (all *p* > 0.05). Looking at the number of streamlines connecting the two hemispheres of the SFG, MFG, and IFG ROIs, a significant effect of reconstruction on the number of MFG streamlines is observed [*F*_(6, 91)_ = 15.216, *p* < 0.001], but no significant effect is found for superior or inferior frontal gyrus streamlines. *Post-hoc* Bonferroni tests demonstrate that the DSI with deconvolution reconstruction produces significantly more MFG streamlines than all other methods (all *p* < 0.05). The DTI, ball-and-sticks (0.01 and 0.03), and GQI reconstructions do not differ significantly in the number of MFG streamlines recovered, nor do the DSI, GQI, and GQI with deconvolution methods, but the mean trends show that the model-free methods all capture more MFG streamlines than the model-based methods (See Table 1). DSI and GQI with deconvolution yield a significantly greater number of MFG streamlines relative to the single- and multi-tensor methods (all *p* < 0.05). Means and standard deviations for the ratio of SFG, MFG, and IFG streamlines resolved with each reconstruction methods are listed in Table 2.

**Table 1:** **Number of streamlines terminating in superior, medial, and inferior frontal gyrus for different reconstruction methods**.

	**Total**		**SFG**		**MFG**		**IFG**	
	***M***	***SD***	***M***	***SD***	***M***	***SD***	***M***	***SD***
DTI	14840.14	2863.46	544.07	389.29	2.50	4.50	74.79	225.90
BSM (0.01)	5763.43	1670.48	418.79	262.11	67.29	54.90	9.29	17.30
BSM (0.03)	9306.36	3045.23	367.36	229.99	65.57	53.23	5.43	9.36
DSI	8642.57	3032.74	549.36	380.63	477.43	401.60	40.64	40.26
DSId	10122.50	2961.93	561.21	368.43	877.00	585.66	79.79	88.68
GQI	7195.14	1955.62	351.00	264.93	258.43	260.74	11.93	24.02
GQId	7843.50	1696.80	334.71	262.45	506.71	263.10	31.64	48.95

**Table 2:** **Ratio of superior, medial, and inferior frontal gyrus streamlines resolved with different reconstruction methods**.

	**% SFG**		**% MFG**		**% IFG**	
	***M***	***SD***	***M***	***SD***	***M***	***SD***
DTI	3.68	2.70	0.02	0.03	0.58	1.74
BSM (0.01)	6.93	3.52	1.35	1.22	0.17	0.34
BSM (0.03)	9.74	1.93	0.82	0.77	0.06	0.11
DSI	7.32	6.46	5.05	3.17	0.43	0.38
DSId	5.92	4.25	8.30	4.92	0.69	0.67
GQI	5.09	3.78	3.41	2.98	0.15	0.32
GQId	4.34	3.24	6.46	3.23	0.42	0.71

**Figure 8:**
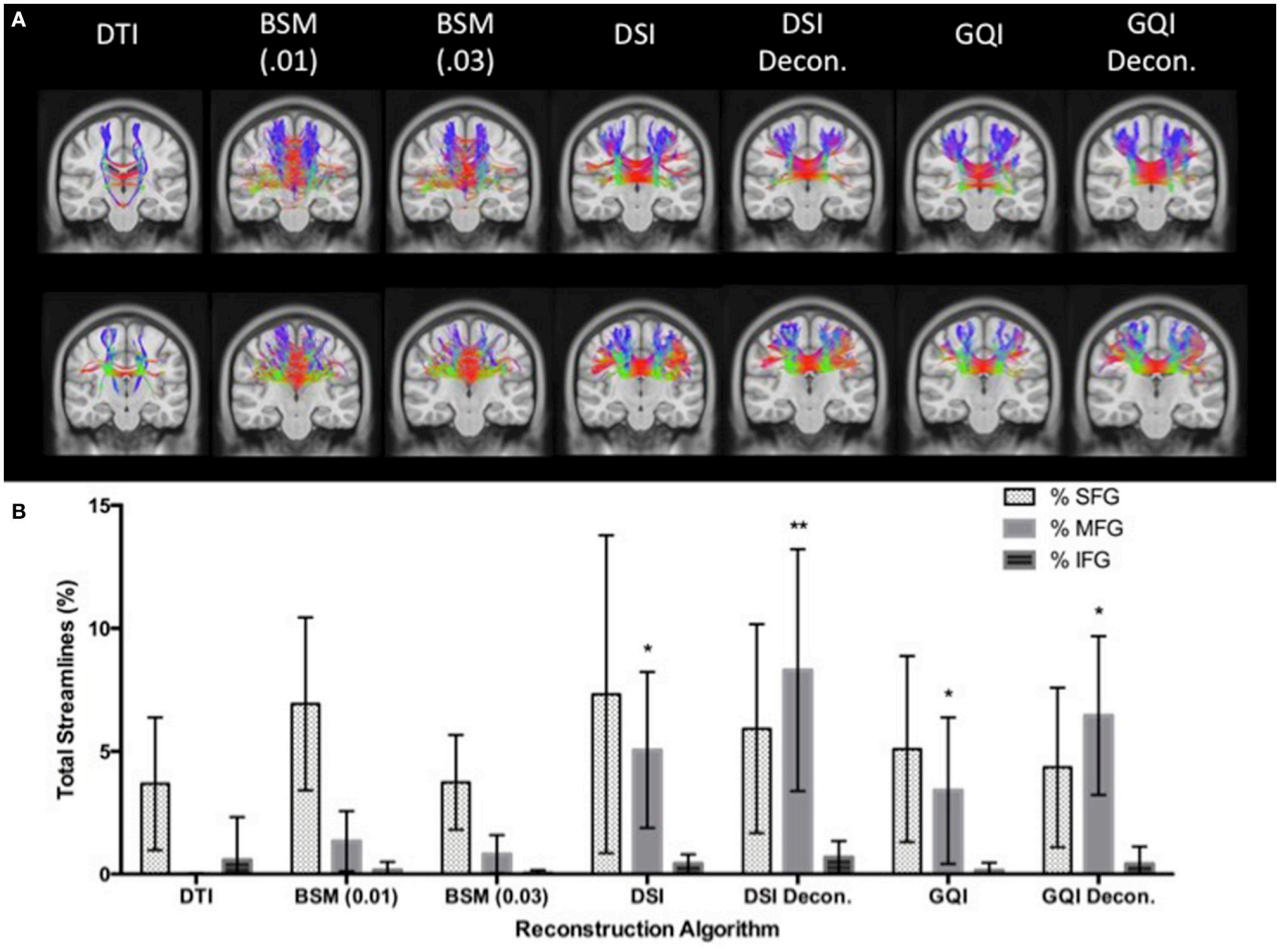
**Human Fiber Crossing Results. (A)** Tractography results of two human participants demonstrate the variations across reconstruction approach in the corpus callosum. **(B)** DTI and ball-and-sticks (0.01 and 0.03) reconstruction methods resolved a smaller percentage of MFG streamlines compared to the GQI and DSI approaches. There was no significant difference between DSI or GQI approaches in the MFG. There were no significant differences found between reconstruction methods for the SFG or IFG. DTI, diffusion tensor imaging; DSI, diffusion spectrum imaging; Decon, deconvolution; GQI, generalized q-sampling imaging; BSM, ball-and-sticks model; SFG, superior frontal gyrus; MFG, medial frontal gyrus; IFG, inferior frontal gyrus. ^*^Significance of at least *p* < 0.05.

A 7 × 3 ANOVA reveals a significant main effect of reconstruction method [*F*_(6, 273)_ = 9.313, *p* < 0.001] and ROI [*F*_(2, 273)_ = 77.2, *p* < 0.001] on the ratio of streamlines relative to the total number of streamlines, as well as a significant reconstruction by ROI interaction [*F*_(12, 273)_ = 5.78, *p* < 0.001]. In order to interpret this interaction, additional ANOVA analyses were performed to look at the effect of reconstruction method on the ratio of streamlines resolved for each ROI separately. These analyses reveal that the effect of reconstruction was only significant for the ratio of MFG streamlines relative to the total number of streamlines. No significant difference is observed between the DTI and ball-and-sticks (0.01 and 0.03 fiber ratio thresholds), but both approaches perform significantly worse than the DSI and GQI reconstructions. Similarly, DSI, DSI with deconvolution and GQI with deconvolution do not differ significantly, but the GQI without deconvolution resolves a significantly smaller proportion of MFG streamlines relative to the DSI with deconvolution reconstruction (Figure 8).

In order to assess the impact of applying a deconvolution algorithm, a 2 × 2 ANOVA was conducted to determine the effect of reconstruction (DSI and GQI only) and deconvolution on the ratio of MFG streamlines resolved. No significant effect of reconstruction method is present, but a significant main effect of deconvolution is observed [*F*_(1, 52)_ = 10.358, *p* < 0.01]. No significant interaction is present, indicating that for both DSI and GQI methods, the application of a deconvolution algorithm increases the amount of MFG streamlines resolved.

## Discussion

The results shown here reject the hypothesis that the current capillary phantom can faithfully provide a performance evaluation consistent with the *in vivo* human study. When benchmarked on regions with similarly complex multiple crossing geometry in both the phantom and human pathways, validation results were highly dependent upon the medium used. Specifically, in the phantom, the model-based ball-and-sticks model outperformed all other approaches for resolving crossing fibers, whereas the model-free methods performed better in the human pathways. The phantom results showed that the ball-and-sticks reconstruction at a 0.01 fiber ratio threshold produces significantly more correct streamlines along the crossing pathway than GQI. Interestingly, DTI also outperformed GQI in the U-fiber region of the phantom. GQI reconstruction underperforms, particularly, in the quantity of streamlines generated. This is evident from the straight arm crossing fiber configuration where both GQI and DTI fail to produce any correct streamlines. The overall phantom results reveal a performance order that a complicated model works better than a simple tensor model, and a simple tensor model was better than model-free methods.

The *in vivo* study showed a completely opposite results. The GQI reconstructions showed marked improvements over the model-based methods for resolving crossing fibers *in vivo*. Both the DSI and GQI methods produced better lateral coverage of the frontal cortex than the DTI or ball-and-sticks reconstructions in human subjects. GQI results are consistent with measurements obtained using the DSI reconstruction within the MFG. The advantage of adding the deconvolution algorithm to DSI and GQI reconstructions is also evident in the *in vivo* results, showing greater lateral coverage in both circumstances. DSI with deconvolution produces a greater ratio of MFG streamlines relative to all other reconstruction methods. More importantly, DTI performed better than a more complicated ball-and-sticks model, and thus the overall results revealed that a model-free method was better than a simple tensor model, and a single tensor model was better than a complicated model.

Thus, there is a substantial discrepancy between the phantom and *in vivo* studies in the performance. A possible explanation for this discrepancy is the design of the diffusion phantom. The DTI and ball-and-sticks approaches use the tensor model that implicitly assumes uniformity of the diffusion pattern in the fiber populations. This assumption fits well with the design of the diffusion phantom, in which, the composition of fiber bundles is constant. The uniformity of fiber populations in typical phantom constructions may explain why the model-based methods are better suited to capture crossing fibers in phantom studies. For example, the ball-and-sticks model assumes that the characteristics of diffusion are identical in each direction. While this matches the phantom configuration, the synthetic fiber bundles in the phantom are not structurally congruent with axons found in the brain due to their homogenous diameters and lengths. The axonal fibers that make up major white matter pathways of the brain are heterogeneous in terms of size and myelination (i.e., Aboitiz et al., 1992). Besides axonal structure, there are additional complexities such as cellular microstructure that may also contribute to the diffusion signal in neural tissue. Recent literature suggests that as much as 40% of a 2.5 mm isotropic voxel, standard for *in vivo* high angular resolution diffusion imaging sequences, is composed of neuroglia such as astrocytes (Walhovd et al., 2014). Therefore, it is likely the simplicity of a diffusion phantom may overestimate the performance of model-based methods such as DTI and ball-and-sticks model.

This conclusion can also be applied to most simulation studies that have also assumed uniformity in the diffusion pattern and thus used a uniform diffusion model to simulate diffusion signals. Therefore, such numerical simulations may also favor model-based methods over model-free methods. Alternatively, more recent simulation approaches, such as tractometer (Côté et al., 2012) and diffantom (Esteban et al., 2016), use real data to create digital phantoms, and may represent a useful alternative to simple capillary phantoms for evaluating different diffusion imaging analysis methods. Future research is necessary to establish whether the performance of different reconstruction methods in more advanced simulated data would accurately predict the relative performance of these reconstruction methods *in vivo*.

Another important issue highlighted in this study is that the model-free methods were underestimated in the capillary phantom. For *in vivo* studies, model-free methods, such as GQI, may be more appropriate than the model-based reconstructions for studying biologically relevant fiber geometry as they use a data-driven approach to identify peaks in the diffusion ODF without imposing *a priori* assumptions about the properties of diffusion in the complex axonal structures. Interestingly, this finding is consistent with the recent result of the ISMRM 2015 tractography competition (http://www.tractometer.org/ismrm_2015_challenge/), in which GQI (ID#03) demonstrated the highest percentage of valid connections over all other model-based methods. Even more surprisingly, complicated modeling methods such as constrained spherical harmonics and ball-and-sticks performed worse than a simple DTI-based tractography. This result strongly suggests that the previous phantom and simulation studies may have overestimated the complicated modeling approach over the simple, robust methodology, leading to a serious doubt on the accuracy and reliability of diffusion MRI tractography (Thomas et al., 2014; Reveley et al., 2015).

The use of different acquisition schemes may also have contributed to the discrepant results obtained with the phantom and human datasets. Nonetheless, previous studies have compared reconstruction methods using different acquisition schemes (i.e., Daducci et al., 2014), and the results of reconstruction methods comparisons are often used to guide the selection of reconstruction approach regardless of the acquisition scheme used. Moreover, these acquisition schemes are often specifically tailored to optimize a specific reconstruction method or analysis approach biasing direct comparison. Future studies should confirm whether reconstruction approaches would also perform differently in phantom and human datasets acquired using the same acquisition scheme. Furthermore, the current analysis focused on the use of different reconstruction methods with a deterministic tractography approach. Future research is necessary to compare the relative performance of model based and model free reconstructions using probabilistic tractography.

The large discrepancy between the phantom and *in vivo* results suggest that more realistic diffusion phantoms are needed to replace the current designs that are composed of a host of synthetically derived materials that often do not exhibit diffusion properties similar to biological tissue. Advances in materials engineering provide promising new methods to generate biomimetic phantoms. For example, Hubbard and colleagues utilized electrospinning methods to develop reproducible diffusion phantoms of tunable fiber diameters (1–20 μM) and geometries (Hubbard et al., 2015). Inhomogeneous fiber size, diameter, and geometry are consistent with neural architecture and would provide a better testing platform for diffusion reconstruction comparisons.

Overall, our results cast doubt on the assumption that diffusion imaging analysis methods perform comparably in capillary phantom and *in vivo* human data. While the use of diffusion phantoms to test novel reconstruction methods provides valuable quantifiable information, it may not be used as a sole methodology to evaluate the performance of a method.

## Conclusions

The hypothesis that diffusion phantoms built with capillary tubes can faithfully reveal the performance of reconstruction in the *in vivo* human study is rejected. Diffusion phantoms built with capillary tubes tend to favor more complicated reconstruction models over a simple tensor model or model-free approaches, while in the *in vivo* human brain model-free approaches tended to come out on top. The current findings highlight the need for more biologically realistic diffusion phantoms or simulation to adequately predict the performance of different methods of analysis in neural tissue and to accurately evaluate the relative strengths and weaknesses of various diffusion imaging methods.

## Author contributions

SL, JB conducted the data analysis and made substantial contributions to the interpretation of the findings, in addition to drafting and revising the manuscript and providing final approval of the version to be published. TV, FY each made substantial contributions to the conception and design, acquisition, analysis and interpretation of the data, as well as revising the manuscript critically for important intellectual content and providing final approval of the version to be published. All authors agree to be accountable for all aspects of the work.

## Funding

Funding was provided in part by the Army Research Laboratory under Cooperative Agreement Number W911NF-10-2-0022, NSF BIGDATA 1247658, NSF GRFP DGE-1247842 and the Multimodal Neuroimaging Training Program (MNTP) at the University of Pittsburgh, in collaboration with Carnegie Mellon University, and with funding from the National Institutes of Health (NIH) (grants R90DA023420 and T90DA022761). The views and conclusions contained in this document are those of the authors and should not be interpreted as representing the official policies, either expressed or implied, of the Army Research Laboratory or the U.S. Government. The U.S. Government is authorized to reproduce and distribute reprints for Government purposes notwithstanding any copyright notation herein.

### Conflict of interest statement

The authors declare that the research was conducted in the absence of any commercial or financial relationships that could be construed as a potential conflict of interest.

## Abbreviations

BSM: ball-and-sticks model
dODF: diffusion orientation distribution function
DSI: diffusion spectrum imaging
DTI: diffusion tensor imaging
GQI: generalized q-sampling imaging
ODF: orientation distribution function.
